# Trans, trans‐2,4‐decadienal, a lipid peroxidation product, aggravates insulin resistance in obese mice by promoting adipose inflammation

**DOI:** 10.1002/fsn3.4273

**Published:** 2024-06-17

**Authors:** Yuanyuan Hu, Xiangbo Zeng, Ying Luo, Xuechen Pei, Dayong Zhou, Beiwei Zhu

**Affiliations:** ^1^ Shenzhen Key Laboratory of Food Nutrition and Health, College of Chemistry and Environmental Engineering Shenzhen University Shenzhen China; ^2^ State Key Laboratory of Marine Food Processing & Safety Control, School of Food Science and Technology Dalian Polytechnic University Dalian China

**Keywords:** adipose inflammation, aldehydes, insulin resistance, lipid peroxidation, M1 macrophage

## Abstract

Peroxidation of polyunsaturated fatty acids results in the creation of numerous α, β‐unsaturated aldehydes, many of which are complicated by the development of diabetes. Trans, trans‐2,4‐decadienal (DDE) is a dietary α, β‐unsaturated aldehyde that is commonly found in food and the environment. However, it is unknown whether DDE exposure has some negative effects on glucose homeostasis and insulin sensitivity. This study investigated the biological effects of long‐term DDE exposure in normal chow diet (NCD)‐fed non‐obese mice and high‐fat diet (HFD)‐fed obese mice. Results showed that oral administration of DDE for 14 weeks did not cause severe toxicity in NCD‐fed non‐obese mice but had significant adverse effects in HFD‐fed obese mice. It was found that DDE exposure caused significant increases in LDL and ALT levels and aggravated glucose intolerance and insulin resistance in obese mice. Moreover, DDE robustly accumulated in adipose tissue and promoted the impairment of the insulin signaling pathway in the adipose tissue of obese mice while not affecting the skeletal muscle or liver. Mechanistically, DDE aggravated adipose tissue inflammation by promoting M1 macrophage accumulation and increasing proinflammatory cytokines in the adipocytes of obese mice, thus leading to impaired systemic insulin resistance. These findings provide crucial insights into the potential health impacts of long‐term DDE exposure.

## Graphical abstract

Oral intake of DDE significantly aggravated glucose intolerance and insulin resistance in HFD‐fed obese mice. Mechanistically, DDE aggravated adipose tissue inflammation by promoting M1‐type macrophage aggregation in the adipocytes of obese mice. These results suggest that DDE aggravates systemic insulin resistance in HFD‐fed obese mice by promoting adipose inflammation.

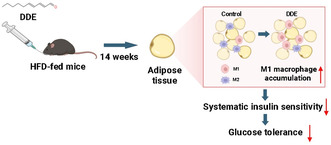

## 1 INTRODUCTION

Polyunsaturated fatty acids (PUFAs) are important nutrients in the diet and are essential for cell function in biological systems; however, they are easily oxidized (Rodriguez‐Amaya & Shahidi, 2021). Peroxidation of PUFAs produces a significant variety of α, β‐unsaturated aldehydes, including 4‐hydroxy‐2‐nonenal (HNE), 4‐hydroxy‐2‐hexenal (HHE), and acrolein (Rodriguez‐Amaya & Shahidi, 2021; Vieira et al., 2017). Increasing evidence indicates that these α, β‐unsaturated aldehydes are implicated in the pathogenesis of insulin resistance and the etiology of type 2 diabetes and its peripheral complications (Jaganjac et al., 2013; Ruskovska & Bernlohr, 2013). It has been found that individuals and animals with diabetes exhibit elevated levels of α, β‐unsaturated aldehydes, and a considerable number of these aldehydes have the potential to impair insulin action, insulin signaling, insulin secretion of beta cells, and insulin‐induced glucose uptake in vitro and in vivo (Jaganjac et al., 2013; Lou et al., 2020; Pillon et al., 2012; Soulage et al., 2018). These results support the potential roles of α, β‐unsaturated aldehydes in the pathogenesis of diabetes.

Trans, trans‐2,4‐decadienal (DDE) is a ubiquitous α, β‐unsaturated aldehyde that forms both endogenously and exogenously from the peroxidation of ω‐6 PUFA (Hu et al., 2021; Wang et al., 2019). It is also used as a flavoring agent and fragrance and is frequently found in food products, especially fried foods (Hu et al., 2021). Meanwhile, DDE is the most prevalent aldehyde in cooking oil fumes (COFs) that have been considered a predominant contributor to COF‐induced lung adenocarcinoma (Chang et al., 2005; Yan et al., 2020). Similar to other α, β‐unsaturated aldehydes, DDE has highly electrophilic properties and can form adducts on biological molecules and activate multiple stress signaling pathways (Hu et al., 2020; Lin et al., 2014). Previous studies have shown that DDE exposure is associated with the pathogenesis of atherosclerosis, colitis, and cancer through mechanisms such as the adduction of protein/DNA and the induction of oxidative stress, inflammation, and mitochondrial damage (Hu et al., 2020; Lin et al., 2014). We recently demonstrated that excess ingestion of DDE induced endothelial dysfunction and elevated blood pressure in rats (Hu et al., 2020). However, whether orally administered DDE has the direct risk of inducing insulin resistance and/or increasing the risk of diabetes in the susceptible population, such as obesity, has not been investigated.

Herein, the purpose of this study was to investigate the impacts of long‐term DDE consumption on glucose tolerance and insulin sensitivity in both normal chow diet‐fed non‐obese mice and high‐fat diet‐fed obese mice and the underlying mechanisms involved. This study adds new evidence for causal links between PUFA peroxidation‐derived α, β‐unsaturated aldehyde exposure and the pathogenesis of diabetes and provides important information to understand the potential health impacts of long‐term DDE exposure.

## 2 MATERIALS AND METHODS

### 2.1 Animals and treatments

A group of eight‐week‐old male C57BL/6 mice was obtained from Changsheng Biotechnology Co., Ltd. and was kept at Dalian Polytechnic University's Animal Cente. All animal experiments were approved by the Institutional Animal Care and Use Committee at Dalian Polytechnic University (LZ0160258). All animals were maintained under standard conditions in a temperature‐controlled room (22 ± 2°C) with a 12 h light/12 h dark cycle and were given ad libitum access to food and water throughout the study. Mice were fed with a high‐fat diet (HFD; 60 kcal% fat, cat# D12492 (Table S1); Research Diets Inc., New Brunswick, NJ, USA) or a standard normal chow diet (NCD; 10% kcal% fat, cat# D12450J (Table S1), Research Diets Inc., USA). They were divided into six groups (*n* = 8 per group) after one week of acclimation: NCD with administration of corn oil, NCD plus administration of 1 mg/kg/day DDE (NCD + DDE1), NCD plus administration of 5 mg/kg/day DDE (NCD + DDE5), HFD with administration of corn oil, HFD plus administration of 1 mg/kg/day DDE (HFD + DDE1), and HFD plus administration of 5 mg/kg/day DDE (HFD + DDE5). The high oral dose of DDE is equivalent to the estimated average human intake level of total unsaturated aldehydes (5 mg/kg body weight per day) (Srivastava et al., 2011). DDE (>98.0%; Macklin Inc., Shanghai, China) was dissolved in fresh corn oil (C8267, Sigma‐Aldrich Co., St. Louis, MO, USA) and administered by intragastric gavage between 8 and 9 a.m. consecutively for 14 weeks. The energy density of corn oil was 8.84 kcal/g, and the lipid composition is shown in Table S2. The mice in the control group were oral gavaged with an equal volume of vehicle (corn oil without DDE). Mouse body weights were monitored weekly, and food intake was monitored biweekly. Blood glucose levels were assessed biweekly after a 4‐h morning fast. Two hours after the last administration, all mice were fasted for 8 h. The mice were anesthetized with isoflurane and then sacrificed to obtain their blood, hearts, livers, spleens, lungs, kidneys, skeletal muscle, and epididymal fat pads. Plasma was obtained by centrifugation from the EDTA tube and then freezing at −80°C. The tissue samples were also frozen at −80°C until further use.

### 2.2 Biochemical determinations

Blood glucose was measured biweekly from whole blood following a 4 h morning fast using a handheld glucometer (Lifescan, Milpitas, CA, USA). Plasma biochemical parameters (total cholesterol (TC), total cholesterol (TG), high‐density lipoprotein (HDL), low‐density lipoprotein (LDL), alanine aminotransferase (ALT), creatinine (Cr), and blood urea nitrogen (BUN)) were determined by the maccura i3000 automatic biochemical analyzer (Maccura Biotechnology Co., Ltd., China). Plasma free fatty acids (FFA) and glycated hemoglobin (GHb) were measured by commercial kits (Jiancheng Bioengineering, China). Plasma fasting blood insulin, leptin, adiponectin, monocyte chemotactic protein 1 (MCP‐1), and tumor necrosis factor alpha (TNF‐α) were determined by ELISA kits (Jiancheng Bioengineering, China). All these indexes were measured following the manufacturer's instructions. The homeostasis model assessment of basal insulin resistance (HOMA‐IR) was calculated as previously described (Srivastava et al., 2011).

### 2.3 Oral glucose tolerance test and insulin tolerance test

After fasting for 8 h, the mice were subjected to an oral glucose tolerance test (OGTT) in week 13. 2 mg/kg of glucose were given to mice orally, and blood was collected after glucose administration. After fasting for 4 h in the morning, an insulin tolerance test (ITT) was carried out at the end of week 14. 0.7 units/kg of insulin was injected into the peritoneal cavity, and blood was collected after the insulin injection. Blood glucose levels were estimated using a commercial glucometer. The integrated area under the response curve (AUC) for glucose was calculated as previously described (Hu et al., 2017).

### 2.4 DDE measurement

DDE was quantified by a liquid chromatography tandem‐mass spectroscopy (LC–MS/MS) assay as previously described (Liu et al., 2020). Briefly, liver, skeletal muscle, and epididymal fat samples were weighed, minced in phosphate buffered saline (PBS), and then centrifuged. 500 μL of the supernatants were treated with 100 μL of 2, 4‐dinitrophenylhydrazine (DNPH) solution (0.05 M in acetonitrile with 0.2% formic acid) at 25°C for 1 h. Then, dichloromethane was used to extract the aldehyde‐DNPH derivatives. After drying under nitrogen, 500 μL of HPLC‐grade acetonitrile was added to redissolve for LC–MS/MS analysis. For the standard, the DDE solution was treated under the same conditions as described above. Samples were filtered through membranes with a pore size of 0.22 μm. The LC analysis was conducted on a Shimadzu LC‐30AVP system (Shimadzu Co., Tokyo, Japan). The MS analysis was performed on an API 5500 Qtrap triple quadrupole mass spectrometer (AB Sciex, Foster City, CA, USA) by using multiple reaction monitoring modes.

### 2.5 Histopathological observation

Epididymal fat samples were fixed, embedded in paraffin, and subjected to hematoxylin and eosin staining for H&E analysis. Slides were observed and captured under an optical microscope. Image‐Pro analysis software was used to uniformly measure the number of adipocytes in three visual fields in each section and the corresponding visual field area (mm^2^), with mm as the standard unit. Five adipocytes were selected for each section to measure adipocyte diameter (mm), and adipocyte density (n/mm^2^) = adipocyte/visual field area was calculated.

### 2.6 Measurement of caspase‐3 activity

Caspase‐3 activity in freshly epididymal fats was determined using a caspase‐3 activity assay kit (Beyotime, China) per the manufacturer's instructions.

### 2.7 Immunohistochemistry

Epididymal fats were fixed, embedded in paraffin, and sectioned. The macrophages were stained with the anti‐F4/80 antibody (Abcam), and the nuclei were stained with hematoxylin. Then the accumulation of macrophages in adipose tissue was assessed using the ratio of the number of F4/80‐positive cells to the number of total cells. The histochemical score (H‐score) was calculated based on the intensity of nuclear staining and the proportion of labeled positive cells (Detre et al., 1995).

### 2.8 Western blot analysis

Liver, skeletal muscle, and epididymal fat pads were homogenized in RIPA buffer (Boster, China). Protein contents were determined by a BCA assay (Beyotime). Equal amounts of proteins (15 μg) were separated by using SDS‐polyacrylamide gel electrophoresis and electrotransferred onto a PVDF membrane. After blocking with 5% milk, the membranes were exposed to primary and secondary antibodies. The blots were then identified using an enhanced chemiluminescent reagent (Thermofisher, China). The primary antibodies used in this study were anti‐phospho‐IRS1 (Ser307; 05‐1087, EMD Millipore), anti‐IRS1 (#2382, Cell Signaling), anti‐phospho‐Akt (Ser473; #4060, Cell Signaling), anti‐Akt (#2920, Cell Signaling), PPARɤ (#sc‐7273, Santa Cruz), and Caspase 3 (#9662, Cell Signaling). The quantification plot was based on densitometry analysis software (Bio‐Rad, Hercules, CA, USA).

### 2.9 Quantitative real‐time PCR

The Trizol reagent was used to isolate RNA from adipose tissue, which was then reverse‐transcribed using a kit from Takara, Japan. Relative gene expression was quantified using the Ct method and a real‐time PCR system. Primer sequences are listed in Table 1.

**TABLE 1:** Primers for qTR‐PCR.

Gene name	Forward primer (5′‐3′)	Reverse primer (5′‐3′)
TNF‐α	CCCTCACACTCAGATCATCTTCT	GCTACGACGTGGGCTACAG
IL‐1β	GCAACTGTTCCTGAACTCAACT	ATCTTTTGGGGTCCGTCAACT
MCP‐1	CCACTCACCTGCTGCTACTCAT	TGGTGATCCTCTTGTAGCTCT
CD11c	ACACAGTGTGCTCCAGTATGA	GCCCAGGGATATGTTCACAGC
F4/80	CTTTGGCTATGGGCTTCCAGTC	GCAAGGAGGACAGAGTTTATCGTG
iNOS	GAGGCCCAGGAGGAGAGAGATCCG	TCCATGCAGACAACCTTGGTGTTG
GAPDH	AGGTCGGTGTGAACGGATTTG	TGTAGACCATGTAGTTGAGGTCA

### 2.10 Statistical analysis

Statistical analysis was carried out with Prism 8.0 software (GraphPad, San Diego, USA). One‐way ANOVA was used to determine statistical significance, followed by Bonferroni's correction. The data is shown as the means ± standard error of the mean (SEM) unless indicated otherwise. *p* < .05 is considered statistically significant.

## 3 RESULTS

### 3.1 Effect of DDE on basic parameters in NCD‐fed non‐obese and HFD‐fed obese mice

**FIGURE 1:**
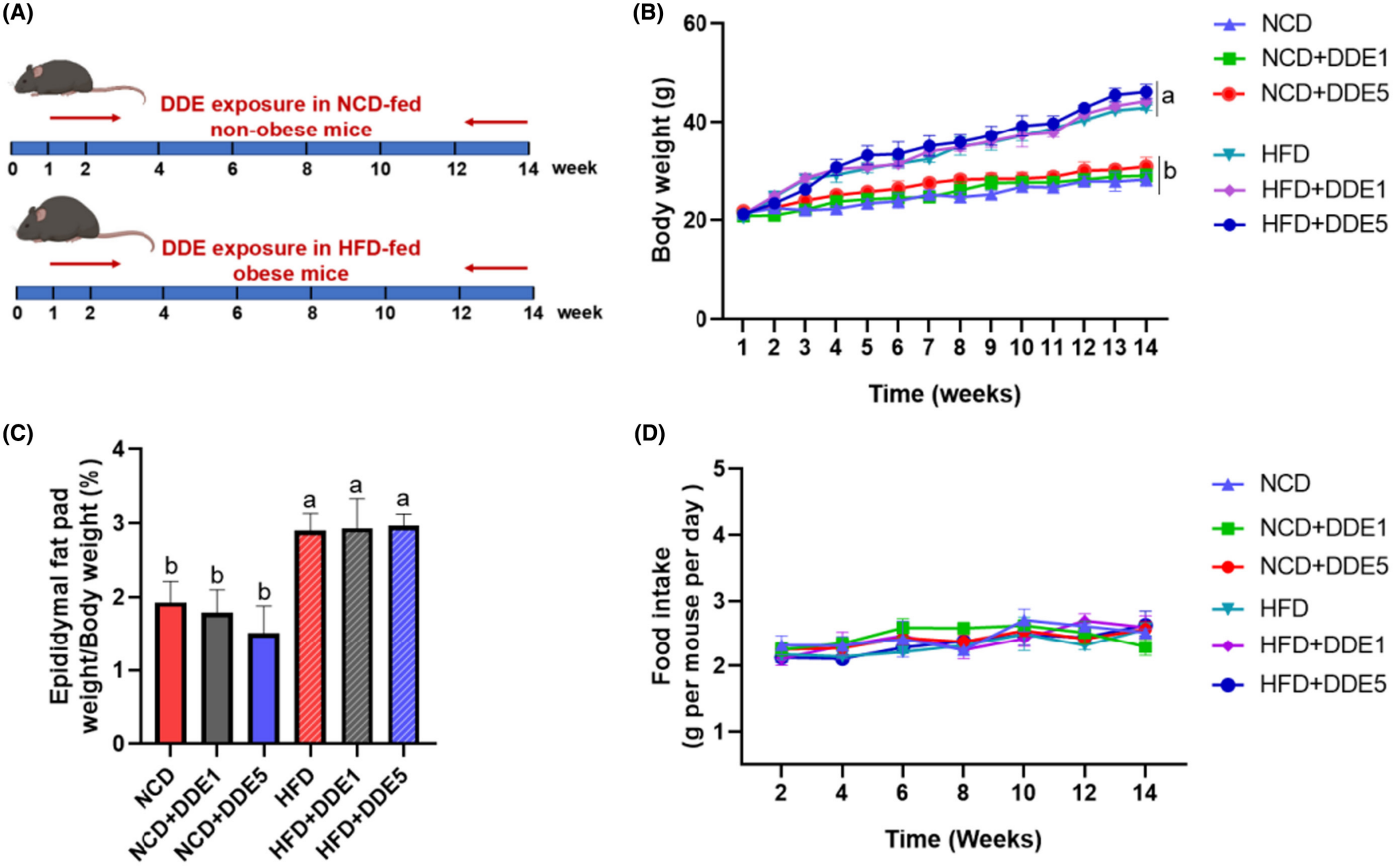
Body weight and food intake of mice after a 14‐week oral exposure to DDE. (A) Schematic representation of the experimental design. (B) Body weight. (C) Percentage of epididymal fat weight to body weight ratio. (D) Food intake. *n* = 6–8. Different letters (a, b) in the panel mean significant differences (*p* < .05).

First, we investigated whether DDE exposure for a continuous 14‐weeks period had any effect on the basic parameters in NCD‐fed non‐obese and HFD‐fed obese mice (Figure 1A). As shown in Figure 1B–D, the HFD‐fed control mice had a 52.16% increase in body weight compared to the NCD‐fed control mice at the end of week 14. DDE treatment showed no significant effect on body weight, epididymal fat pad weight, and food intake in both NCD‐fed mice and HFD‐fed mice. Moreover, DDE treatment showed no significant effect on the organ coefficients in both NCD‐fed mice and HFD‐fed mice (Table 2).

**TABLE 2:** Organ coefficients of mice after a 14‐week oral exposure to DDE.

Group	Heart	Thymus	Liver	Spleen	Lung	Kidney	Testes
NCD	0.55 ± 0.05	0.39 ± 0.11	4.23 ± 0.50^a^	0.28 ± 0.02	0.62 ± 0.09	1.41 ± 0.07^a^	0.77 ± 0.12
NCD + DDE1	0.57 ± 0.12	0.41 ± 0.11	4.33 ± 0.32^a^	0.28 ± 0.02	0.66 ± 0.04	1.33 ± 0.05^ab^	0.77 ± 0.22
NCD + DDE5	0.54 ± 0.05	0.34 ± 0.06	4.20 ± 0.29^a^	0.33 ± 0.12	0.63 ± 0.10	1.37 ± 0.04^ab^	0.78 ± 0.15
HFD	0.49 ± 0.02	0.28 ± 0.04	3.38 ± 0.26^b^	0.27 ± 0.03	0.55 ± 0.04	1.17 ± 0.08^b^	0.55 ± 0.06
HFD + DDE1	0.47 ± 0.03	0.29 ± 0.06	3.93 ± 0.08^ab^	0.34 ± 0.04	0.55 ± 0.12	1.27 ± 0.07^ab^	0.61 ± 0.08
HFD + DDE5	0.48 ± 0.02	0.27 ± 0.09	3.41 ± 0.0^9b^	0.36 ± 0.05	0.61 ± 0.12	1.29 ± 0.10^ab^	0.73 ± 0.03

*Note*: Data are expressed as mean ± *SD*, *n* = 6–8. Values with different letters in the same column are significantly different from each other (*p* < .05).

**FIGURE 2:**
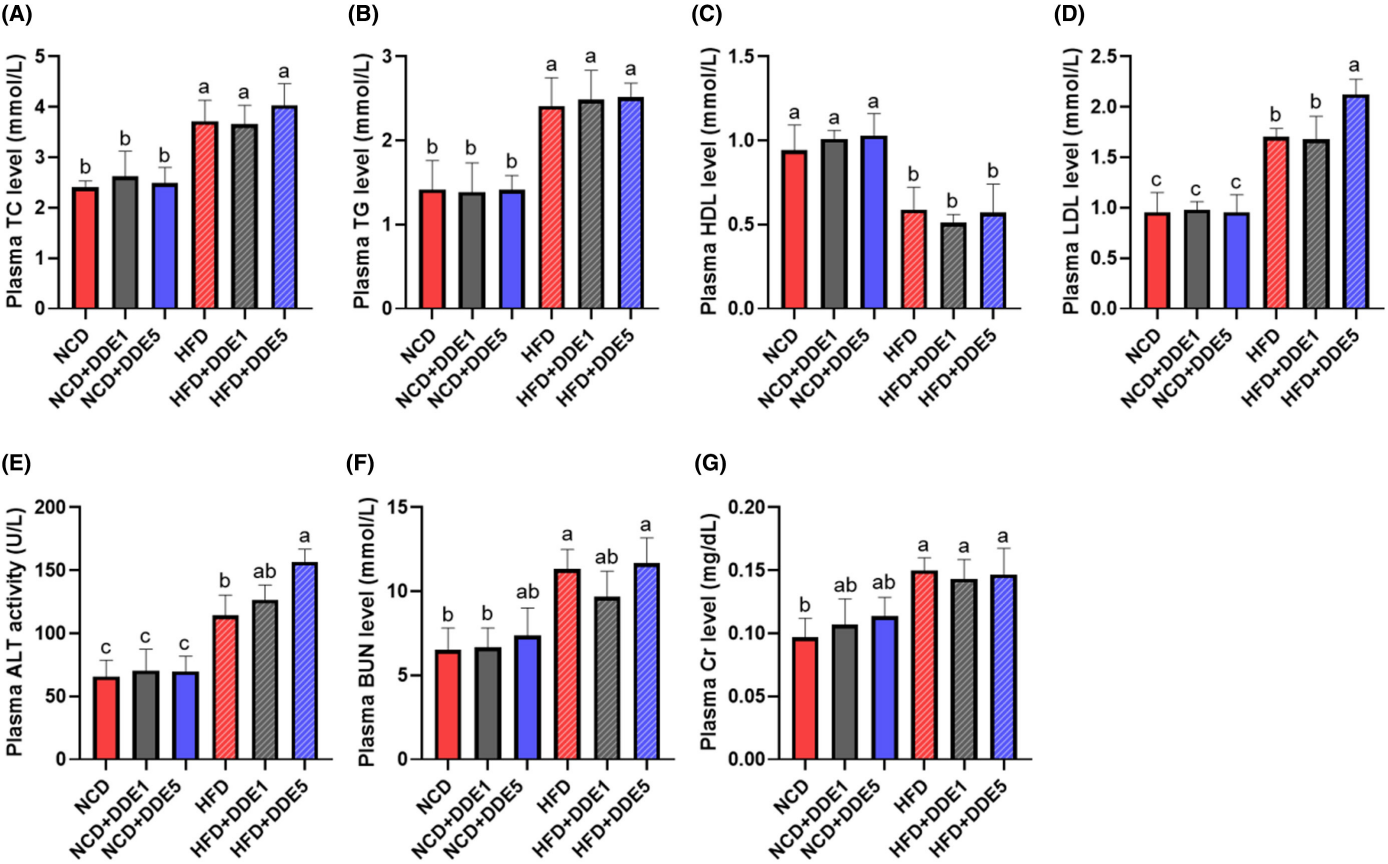
Effects of DDE on plasma biochemical parameters in mice. (A) Plasma total cholesterol (TC) level, (B) Plasma total cholesterol (TG) level, (C) Plasma high‐density lipoprotein (HDL) level, (D) Plasma low‐density lipoprotein (LDL) level, (E) Plasma alanine aminotransferase (ALT) level, (F) Plasma blood urea nitrogen (BUN) level, (G) Plasma creatinine (Cr) level. *n* = 6–8. Different letters (a–c) in the panel mean significant differences (*p* < .05).

To further assess the potential negative impacts of DDE exposure, the plasma biological parameters of mice were assayed. As shown in Figure 2A–G, DDE had no significant effect on plasma LDL, HDL, TG, TC, ALT, BUN, and Cr in NCD‐fed mice, but the high dose of DDE treatment caused significant increases in LDL and ALT levels in HFD‐fed mice (*p* < .05). Notably, in general, the effects of DDE on biochemical parameters were more noticeable in mice fed an HDF diet compared to those fed an NCD diet (Figure 2A–G). These findings indicated that 14 weeks of DDE exposure had a stronger negative effect on HFD‐fed obese mice than on NCD‐fed non‐obese mice.

### 3.2 DDE aggravated glucose intolerance and insulin resistance in HFD‐fed obese mice

**FIGURE 3:**
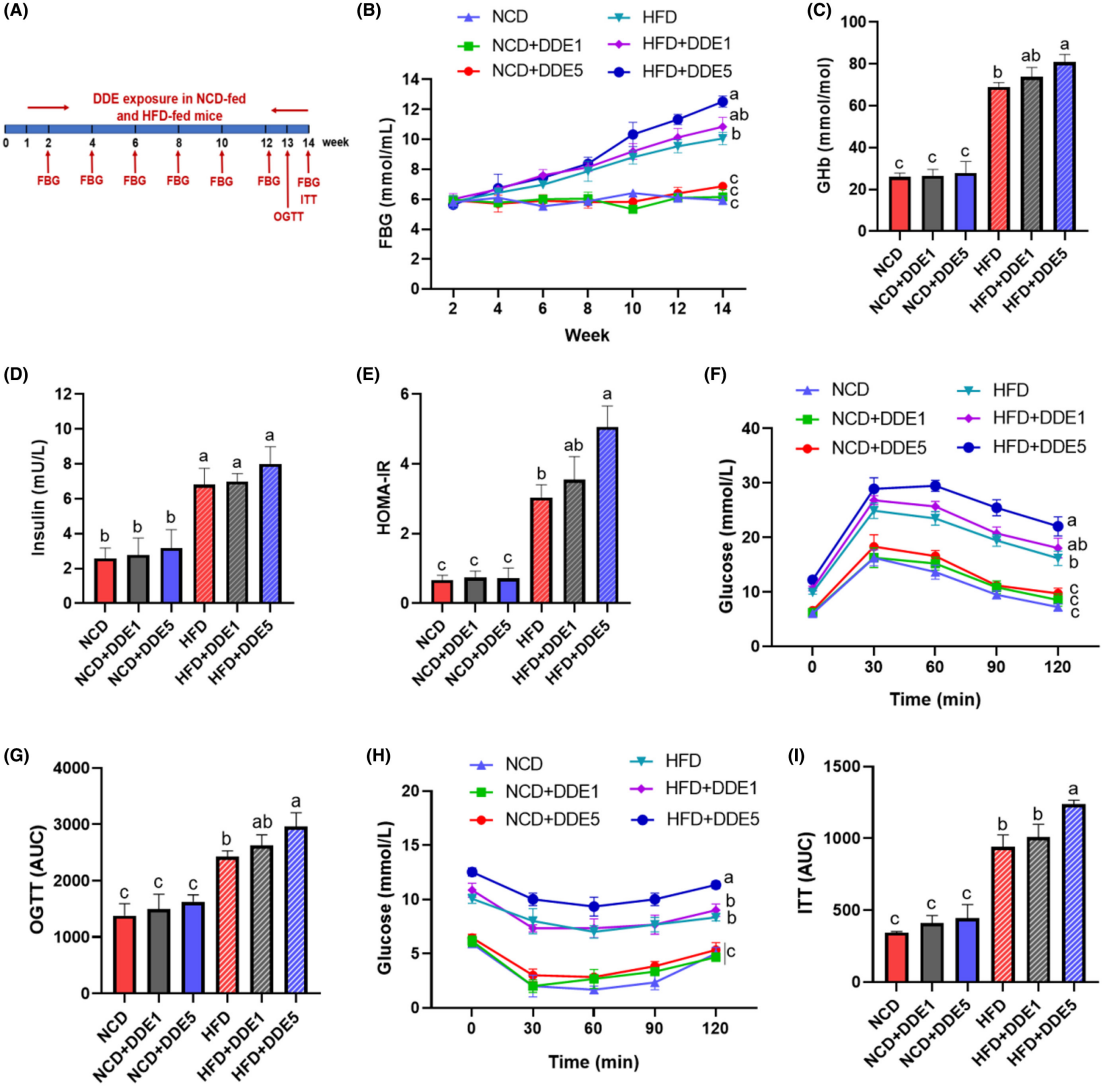
Effects of DDE on glucose tolerance and insulin sensitivity in mice. (A) Schematic representation of the experimental design. (B) Fasting blood glucose levels. (C) Plasma glycated hemoglobin (GHb) level. (D) Plasma insulin levels. (E) The HOMA‐IR index was calculated based on fasting glucose and insulin. (F) Oral glucose tolerance tests (OGTT) in mice after a 13‐week exposure to DDE. (G) The area under the curve (AUC) of OGTT was quantified. (H) Insulin tolerance test (ITT) in mice after a 14‐week exposure to DDE. (I) The area under the curve (AUC) of ITT was quantified. *n* = 6–8. Different letters (a–c) in the panel mean significant differences (*p* < .05).

Insulin resistance is a crucial contributor to the occurrence of type 2 diabetes mellitus (Ying et al., 2017). To investigate whether DDE exposure has detrimental effects on insulin resistance, glucose homeostasis and insulin sensitivity were determined (Figure 3A). As shown in Figure 3B, during the 14‐week feeding trial, DDE treatment showed no significant effect on FBG level in NCD‐fed mice. HFD‐fed mice displayed noticeably higher FBG levels than NCD‐fed mice from week 6. Surprisingly, 5 mg/kg of DDE exposure increased the FBG level in HFD‐fed mice from week 10 to week 14. Furthermore, we found that DDE had no significant effects on plasma GHb (Figure 3C), insulin (Figure 3D), and HOMA‐IR (Figure 3E) in NCD‐fed mice at the termination of 14 weeks, while leading to significantly higher GHb levels (Figure 3C) and HOMA‐IR (Figure 3E) values in HFD‐fed mice compared to the corresponding HFD‐fed control mice. This evidence indicated that 5 mg/kg of DDE exposure impaired systemic insulin sensitivity in HFD‐fed obese mice.

To confirm the effect of DDE exposure on glucose tolerance, OGTT was measured at the end of week 13. Results showed that the high dose of DDE treatment induced a slight impairment of OGTT in NCD‐fed mice (Figure 3F). Notably, DDE treatment exacerbated the HFD‐induced impairment of OGTT in mice, resulting in higher plasma glucose levels during the OGTT study period than those of the HFD control group. The total AUC of glucose concentration in the mice treated with DDE was increased by 21.74% relative to that of HFD control mice (Figure 3G). This result indicated that DDE exposure aggravated HFD‐induced glucose intolerance in HFD‐fed obese mice. To further assess the effect of DDE on insulin action, we also performed an ITT at the end of week 14. As shown in Figure 3H,I, DDE had no significant effect on glucose concentrations during the study period of ITT in NCD‐fed mice, while significantly aggravated HFD‐induced impairment of ITT in HFD‐fed mice, resulting in a 31.04% increase in total AUC compared with the corresponding HFD‐fed control mice. These results together indicated that chronic intake of 5 mg/kg of DDE enhanced glucose tolerance and insulin resistance in HFD‐fed obese mice. Thus, the following studies were only made for the selected groups, namely NCD, NCD + DDE5, HFD, and HFD + DDE5.

### 3.3 DDE impaired insulin signaling pathway in adipose tissue of HFD‐fed obese mice

**FIGURE 4:**
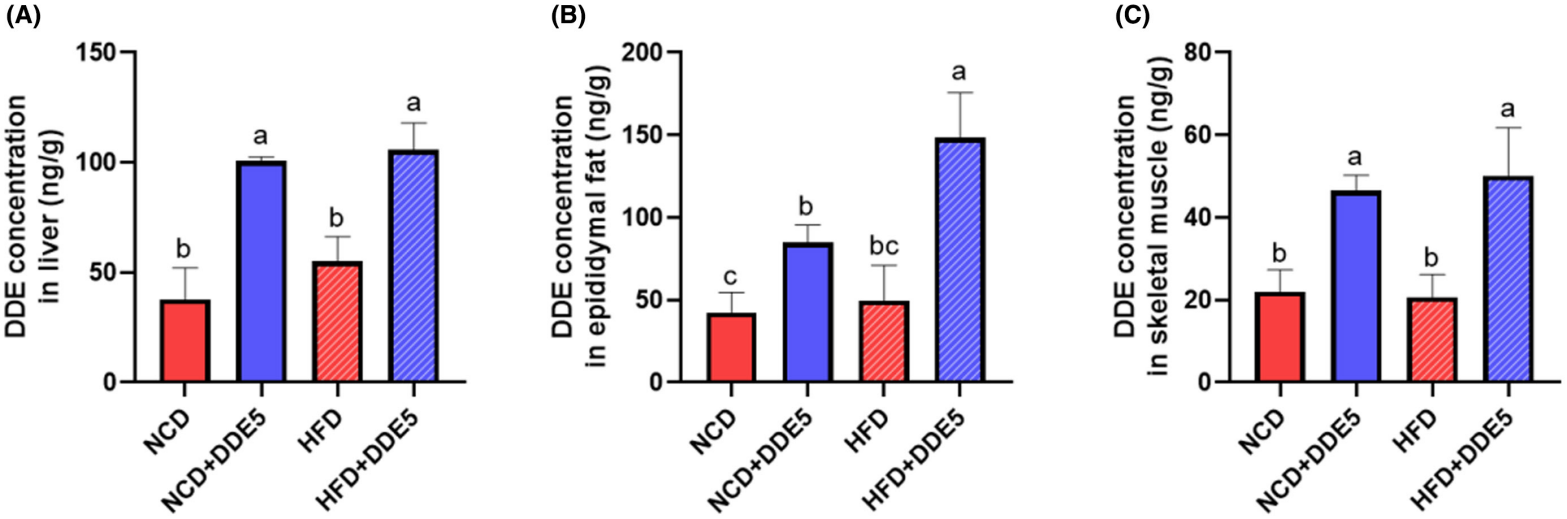
DDE concentrations in different tissues of mice. (A) DDE concentrations in the samples of the liver. (B) DDE concentrations in the samples of epididymal fat. (C) DDE concentrations in the samples of skeletal muscle. *n* = 6–8. Different letters (a–c) in the panel mean significant differences (*p* < .05).

**FIGURE 5:**
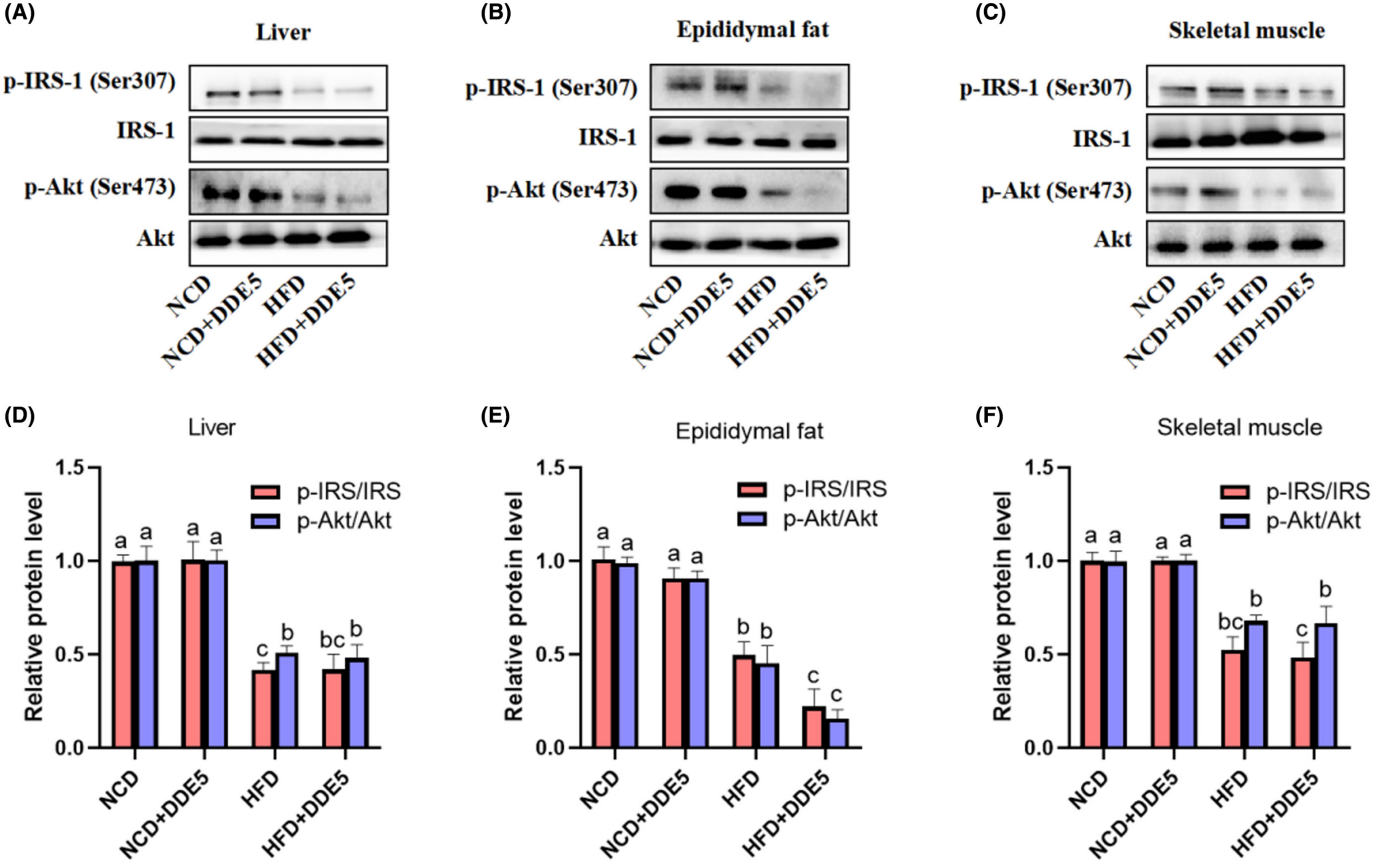
Effects of DDE on the insulin signaling pathway in mice. Representative western blot images of the insulin signal pathway in (A) liver, (B) adipose tissue, and (C) skeletal muscle. Densitometric quantification of p‐Akt^Ser473^ and p‐IRS‐1^Ser307^ levels in (D) liver, (E) adipose tissue, and (F) skeletal muscle, *n* = 3. Different letters (a–c) in the panel mean significant differences (*p* < .05).

To gain insight into how DDE affects insulin resistance, the main target organs of insulin (adipose, liver, and skeletal muscle) were harvested. We first determined whether and how much DDE reaches the main target organs of insulin in mice. The accumulation of DDE in adipose was significantly higher than that in liver and skeletal muscle, indicating that DDE may be more stable in adipose than in classical target tissues (Figure 4A–C). In particular, DDE concentrations in adipose tissue of HFD‐fed mice were roughly 1.4 times higher than the average DDE concentration in the liver and 2.9 times higher than that in the skeletal muscle (Figure 4A–C). Consequently, the consequences of DDE on the insulin signaling pathway in adipose, liver, and skeletal muscle were further explored. As seen in Figure 5A–F, DDE treatment had no evident effect on IRS‐1 phosphorylation and Akt phosphorylation in the liver, adipose, and skeletal muscle of NCD‐fed mice. Notably, adipose tissue in HFD‐fed obese mice showed reduced levels of IRS‐1 and Akt phosphorylation following DDE treatment, while skeletal muscle and liver remained unchanged. These results demonstrated lower insulin sensitivity in DDE‐treated adipose tissue than in the corresponding HFD‐fed control mice, suggesting that DDE promoted the impairment of the insulin signaling pathway in HFD‐fed obese mice. The results were in line with the above and indicated that DDE‐induced impairment of insulin resistance might have a close relationship with adipose tissue.

### 3.4 DDE impaired adipocyte function in HFD‐fed obese mice

**FIGURE 6:**
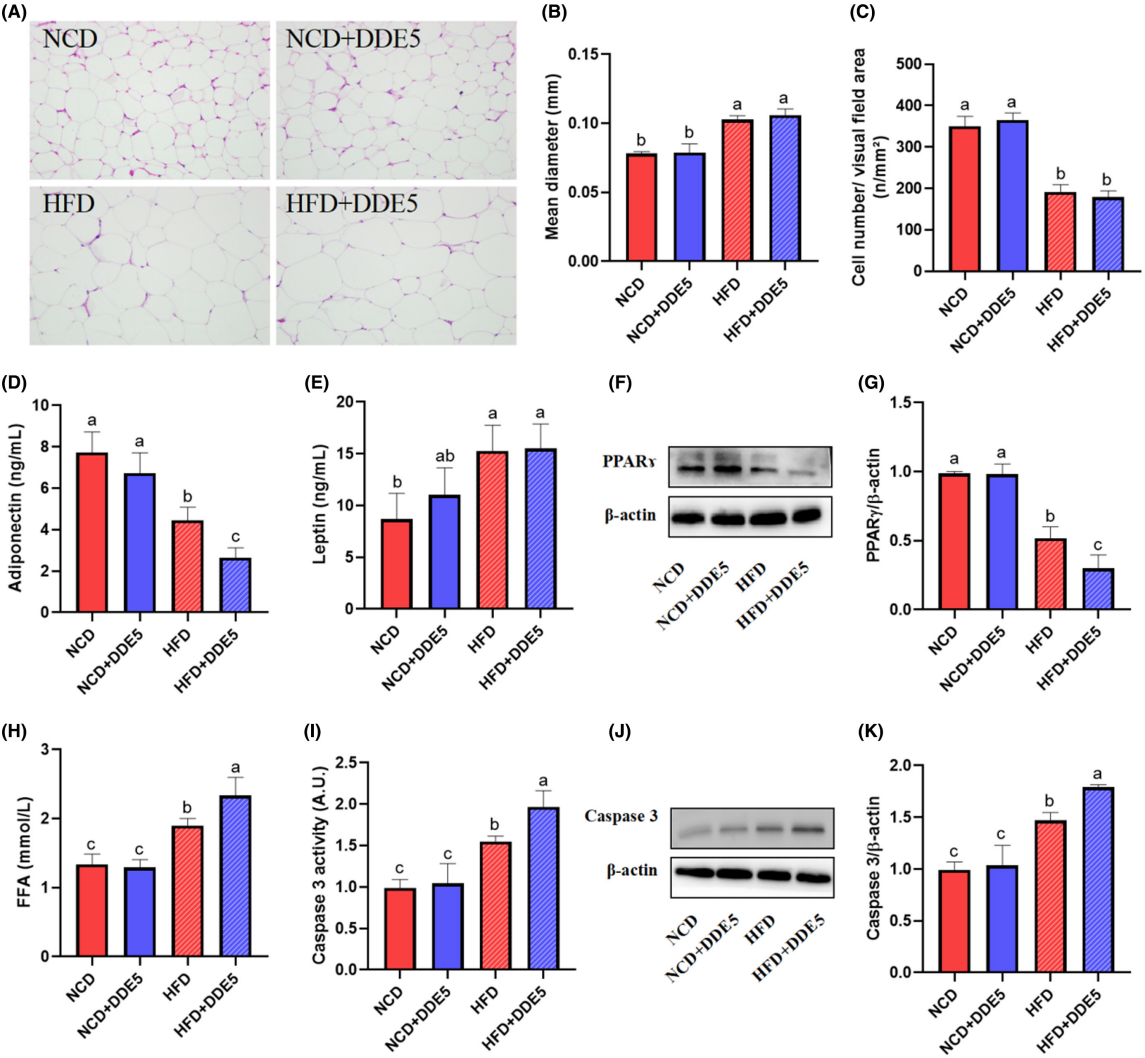
Effects of DDE on adipocyte function and adipogenesis in mice. (A) Hematoxylin and eosin (H&E) staining of epididymal fat; scale bar, 100 μM. (B) The mean diameter of adipocytes in mice. (C) Quantification of adipocyte number per area. (D) Plasma adiponectin level. (E) Plasma leptin level. (F) Representative western blot images of PPARɤ expression in epididymal fat. (G) Densitometric quantification of PPARɤ level. (H) Plasma free fatty acid (FFA) levels. (I) Caspase‐3 activity in epididymal fat. (J) Representative western blot images of caspase 3 in epididymal fat. (K) Densitometric quantification of caspase 3 levels. *n* = 3 mice/group in F, G, J, and K, and *n* = 6–8 mice/group in A–E, H, and I. Different letters (a–c) in the panel mean significant differences (*p* < .05).

Obesity‐related insulin resistance is thought to be primarily caused by adipose tissue dysfunction (Ying et al., 2017). To further investigate the physiological mechanisms underlying the impairment of insulin sensitivity in adipose tissue, the effects of DDE on adipocyte architecture and function were determined. H&E staining of epididymal adipose tissue showed that DDE treatment had no significant effect on adipocyte architecture, adipocyte size, and adipocyte number in both NCD‐fed non‐obese mice and HFD‐fed obese mice (Figure 6A–C). Then we measured the plasma adipokine levels (adiponectin and leptin) secreted by adipose tissue. As shown in Figure 6D,E, DDE treatment showed no significant effects on adiponectin and leptin levels in NCD‐fed mice while inducing significantly lower adiponectin levels in HFD‐fed mice, which favors reduced insulin sensitivity. PPARɤ regulates lipid metabolism and stimulates adipocyte differentiation, which improves blood glucose uptake and insulin sensitivity (El Ouarrat et al., 2020). As shown in Figure 6F,G, the PPARɤ expression was decreased by 41.93% in the adipose tissue of DDE‐treated mice compared with the corresponding HFD‐fed control mice. Insulin promotes the storage of fatty acids as TG and inhibits adipocyte lipolysis (El Ouarrat et al., 2020). As shown in Figure 6H, the plasma FFA level was higher in HFD‐fed mice than the NCD‐fed mice, and DDE exposure significantly increased the FFA level in HFD‐fed mice, suggesting a higher rate of lipolysis in epididymal fat. Additionally, the adipose tissue of mice on a high‐fat diet exhibited considerably higher levels of caspase‐3 expression and activity, in comparison to the NCD‐fed mice (Figure 6I–K), and DDE treatment increased the caspase 3 activity by 21.77% in HFD‐fed mice (Figure 6I), suggesting increased adipocyte apoptosis. Together, these results suggested that DDE exposure induced abnormal secretion of adipokines, reduced PPRAɤ, and increased adipogenesis and adipocyte apoptosis in HFD‐fed obese mice.

### 3.5 DDE increased M1 macrophage accumulation and inflammation in the adipose tissue of HFD‐fed obese mice

**FIGURE 7:**
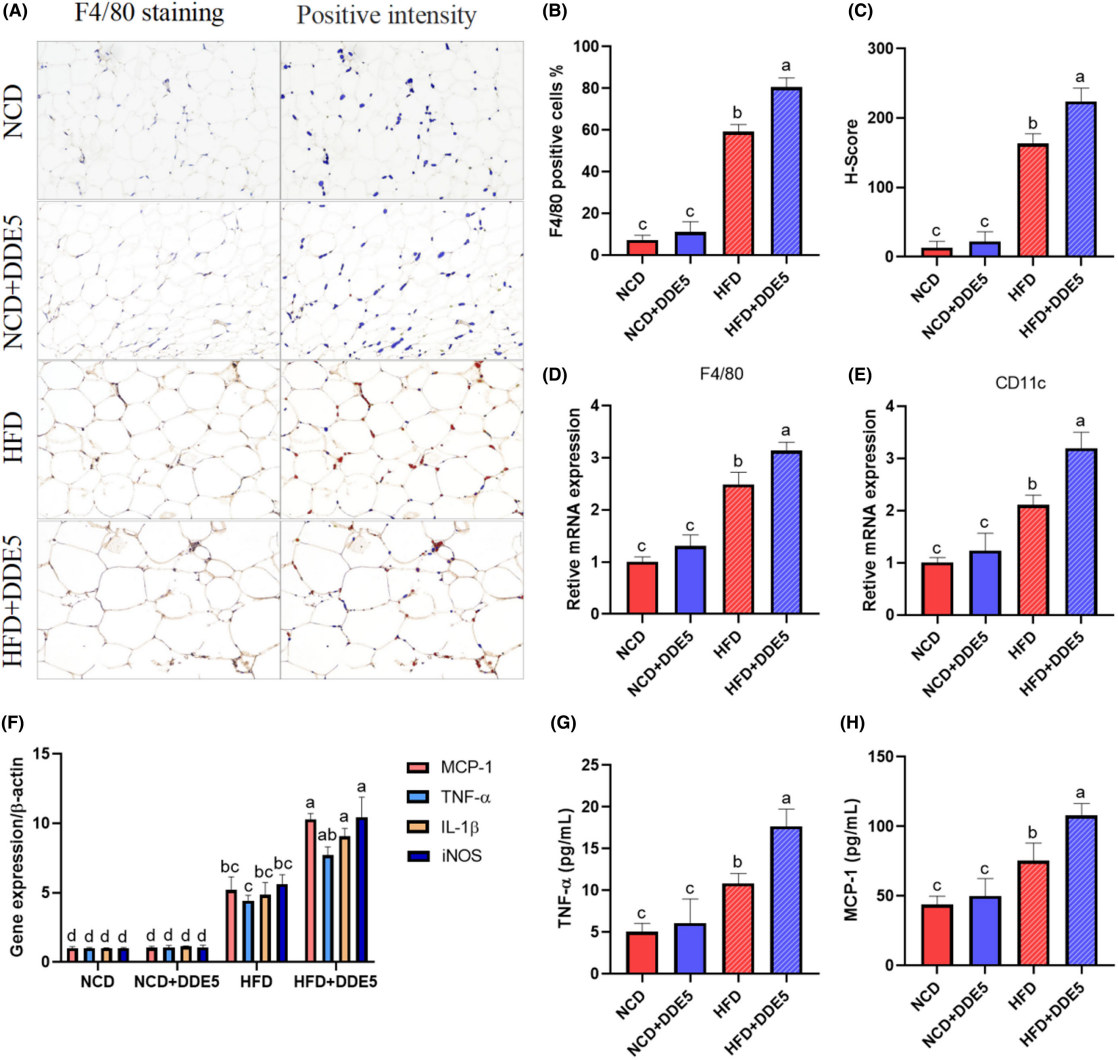
Effects of DDE on adipose tissue inflammation in mice. (A) F4/80 immunostaining in epididymal fat; scale bar, 100 μM. (B) F4/80 positive analysis. (C) H‐Score. (D) Relative mRNA levels of F4/80 in epididymal fat. (E) Relative mRNA levels of CD11c in epididymal fat. (F) Relative mRNA levels of pro‐inflammatory cytokines in epididymal fat. (G) Plasma circulating levels of TNF‐α. (H) Plasma circulating levels of MCP‐1. *n* = 6–8. Different letters (a–d) in the panel mean significant differences (*p* < .05).

The adipose tissue of obese individuals is a breeding ground for macrophages, which play a significant role in the development of chronic tissue inflammation and a subsequent decline in insulin sensitivity (McArdle et al., 2013). Compared to NCD‐fed mice, the epididymal fat of HFD‐fed mice showed significantly increased accumulation of F4/80‐positive macrophages and crown‐like structures (Figure 7A–C). Notably, exposure to DDE resulted in a significant elevation of macrophage accumulation and F4/80‐positive cells in the adipose tissue of mice following a HFD diet (Figure 7A–C). Adipose tissue is home to several subpopulations of macrophages, and M1‐like macrophages are considered to be highly pro‐inflammatory (Shimobayashi et al., 2018). The mRNA levels of F4/80 and CD11c (a known indicator of M1‐like macrophages) in DDE‐treated mice were not significantly changed compared to the corresponding NCD‐fed mice (Figure 7D,E). However, exposure to DDE significantly increased the F4/80 and CD11c mRNA levels in mice on a high‐fat diet, consistent with the findings from immunohistochemical staining (Figure 7D,E).

Additionally, we assessed the mRNA levels of pro‐inflammatory cytokines (iNOS, TNF‐α, IL‐1β, and MCP‐1) in the adipose tissue of mice, all of which are known to contribute to insulin resistance (Unamuno et al., 2018). The NCD‐fed mice exhibited lower mRNA expressions of inflammatory cytokine levels in epididymal fat compared to HFD‐fed mice. DDE treatment leads to higher inflammatory cytokine levels in the HFD‐fed mice (Figure 7F). Consistently, the plasma circulating levels of TNF‐α and MCP‐1 in the mice treated with DDE were increased by 63.08% and 43.09%, respectively, relative to those of HFD‐fed control mice (Figure 7G,H). The findings indicated that exposure to DDE promoted the buildup of M1 macrophages and the secretion of inflammatory cytokines by adipocytes in adipose tissue, ultimately leading to a decrease in insulin sensitivity.

## 4 DISCUSSION

In this study, we demonstrated that oral intake of DDE for 14 weeks significantly aggravated glucose intolerance and insulin resistance in HFD‐fed obese mice while having no significant adverse effect in NCD‐fed non‐obese mice. We also showed that DDE robustly accumulated in adipose tissue, and aggravated the impairment of adipose insulin sensitivity and adipocyte dysfunction in obese mice. Moreover, DDE exposure induced adipose tissue inflammation by inducing M1‐type macrophage aggregation and increasing proinflammatory cytokines in the adipocytes of obese mice. These results indicated that DDE aggravated systematic insulin resistance in obese mice by promoting adipose inflammation. This study provides essential knowledge on the potential health threats posed by long‐term DDE exposure.

In recent years, ubiquitous chemicals in food processing, preservation, and packaging of foods, as well as chemicals that exist in the environment, have attracted increasing attention for being considered as possible causal agents of developing diabetes (Sánchez‐Tapia et al., 2020). Aldehydes are highly reactive carbonyl chemicals that are widely present in foods, especially processed foods. Till now, more than 300 different types of aldehydes have been identified in different foods or food components (Conklin et al., 2010; Guillén & Goicoechea, 2008). One of the main aldehydes resulting from lipid oxidation is DDE, which is widely present in the environment and can frequently be found in food and water, posing potential harm (Hu et al., 2020). In this study, we comprehensively compared the physiological effects of DDE exposure on both NCD‐fed non‐obese mice and HFD‐fed obese mice. The results showed that DDE exposure for 14 weeks had no significant toxic effects on NCD‐fed non‐obese mice but significantly increased the LDL and ALT levels in HFD‐fed obese mice. These results suggest that DDE may induce dyslipidemia and a possible mild adverse effect in the liver of obese mice. Insulin resistance, commonly found in obese individuals, is a major underlying cause of metabolic disorders and other chronic diseases, ultimately leading to the development of type 2 diabetes (El Ouarrat et al., 2020; Liang et al., 2019). It causes the insulin target organs to be less sensitive to insulin action, making normal levels of insulin ineffective in lowering blood glucose and leading to hyperglycemia (El Ouarrat et al., 2020). In this study, HFD‐fed obese mice had significantly higher FBG and insulin levels, as well as HOMA‐IR values, than NCD‐fed non‐obese mice, suggesting the occurrence of glucose intolerance and insulin resistance. The impairment of OGTT and ITT in mice further demonstrated this phenomenon. Interestingly, we found that orally administered DDE for 14 weeks aggravated HFD‐induced glucose intolerance and insulin resistance in obese mice while having no significant effect on non‐obese mice, suggesting that DDE may increase the risk of diabetes in the susceptible population, such as obesity. In this regard, it has been reported that obese populations might be more susceptible to the potentially harmful effects of diet, environment, and other factors (Cao et al., 2020). Consistently, previous studies have reported that exposure to propionate (Tirosh et al., 2019), phosphates (Uribarri & Calvo, 2014), and sweeteners (Jimenez‐Gomez et al., 2013; Liang et al., 2019) may aggravate HFD‐induced insulin resistance.

IRS‐1, a key component of insulin receptor kinase, is indispensable for the activation of downstream metabolism (Ying et al., 2017). In the insulin signaling pathway, Akt phosphorylation serves as a pivotal mechanism in facilitating glucose uptake and glycogen synthesis while also actively regulating glycogen synthase activity to maintain glucose balance (Hou et al., 2017; Sharma et al., 2023). Consistent with previous studies, HFD significantly decreased phosphorylation of IRS‐1 and Akt in the adipose, liver, and skeletal muscle of mice. DDE preferentially accumulated in adipose tissue and further exacerbated the HFD‐triggered decline in IRS‐1 and Akt phosphorylation in obese mice, while this effect was not observed in skeletal muscle and liver. In line with this, HNE, which has a similar chemical structure and reactivity to DDE, was reported to impair the ability of insulin to stimulate glucose uptake in mice as well as in adipocyte cells (Soulage et al., 2018). These results indicated that long‐term DDE exposure promoted obesity‐induced impairments of the IRS‐1/Akt signaling pathway in adipose tissue, thus inducing systemic insulin resistance.

Adipose tissue is both an energy warehouse for storing TG and an active endocrine organ, participating in metabolic regulation through the release of a variety of adipokines, including leptin, adiponectin, and TNF‐α (Rosen & Spiegelman, 2006). Adiponectin is an insulin‐sensitizing adipokine and is also an important factor in regulating blood glucose homeostasis and lipid metabolism (Li et al., 2021). In this study, it was found that DDE treatment significantly decreased the plasma adiponectin level in HFD‐induced obese mice. PPARɤ, a transcription factor, is essential in regulating lipid metabolism and influencing the function of adipose tissue in whole‐body glucose metabolism (Rosen & Spiegelman, 2006). The present study showed that PPARɤ expression in DDE‐treated obese mice was significantly lower in adipose tissue than in the obese control mice. Insulin can generally promote fat synthesis and storage, reduce free fatty acids in the blood, and inhibit fat decomposition and oxidation (El Ouarrat et al., 2020). This study showed that DDE‐treated mice had notably elevated plasma FFA levels compared to the control mice fed with a high‐fat diet, suggesting a higher rate of lipolysis in epididymal fat. Additionally, DDE induced an increase in HFD‐induced apoptosis in adipose tissue, as measured by caspase activity. In agreement with the present results, HNE impaired adipose tissue function, leading to the loss of function of key proteins related to lipid metabolism and impaired insulin action in adipocytes (Jaganjac et al., 2013). Overall, these results demonstrated that DDE exposure exacerbated obesity‐related adipocyte dysfunction, possibly contributing to DDE‐induced insulin resistance.

Inflammation in adipose tissue greatly contributes to the onset of obesity and insulin resistance, leading to insulin resistance both locally and systemically (Escalante‐Aburto et al., 2023; Sun et al., 2016). Macrophages in adipose tissue are thought to be a major source of local or systemic inflammation‐mediating factors and are an important mechanism of insulin resistance in obesity (McArdle et al., 2013). M1 macrophages play a crucial role in tissue homeostasis as well as in inflammation and disease progression (Rajasekaran et al., 2019). To further understand the underlying mechanisms responsible for the effects of DDE on adipose insulin sensitivity, we determined the effect of DDE on macrophage infiltration. Our results showed that DDE treatment significantly aggravated the adipose tissue M1 macrophage activation and accumulation in HFD‐fed mice. MCP‐1 is an important chemotactic agent of macrophages for dead adipocytes. MCP‐1 can accelerate adipose tissue inflammation and directly affect adipocyte function and insulin sensitivity (Nishimoto et al., 2016; Ying et al., 2019). Furthermore, the administration of DDE resulted in elevated M1 buildup and a notable increase in the transcription of pro‐inflammatory cytokines in the adipose tissue, ultimately resulting in elevated MCP‐1 and TNF‐α levels in high‐fat diet‐fed mice. Consistently, previous studies show that DDE stimulates the secretion of inflammatory cytokines in macrophages and epithelial cells (Chang et al., 2005; Girona et al., 2001). Collectively, these findings suggested that DDE exposure induced adipose tissue inflammation by promoting the aggregation of M1‐type macrophages and increasing the production of proinflammatory cytokines in adipocytes of obese mice, ultimately leading to impaired insulin sensitivity and glucose tolerance.

In summary, our data demonstrated that DDE aggravated the impairment of systemic insulin resistance in HFD‐fed obese mice by promoting adipose tissue inflammation. This was accompanied by a decrease in adipose insulin sensitivity and an elevation in adipocyte dysfunction and macrophage accumulation. Our findings suggest that long‐term DDE exposure may be a potential risk factor for the development of diabetes in obese humans. These results offer important scientific information for understanding the biological effects and mechanisms of long‐term exposure to PUFA peroxidation‐derived aldehydes.

## AUTHOR CONTRIBUTIONS

**Yuanyuan Hu:** Conceptualization (equal); data curation (lead); investigation (equal); visualization (equal); writing – original draft (lead). **Xiangbo Zeng:** Investigation (equal); methodology (lead); visualization (supporting). **Ying Luo:** Investigation (equal). **Dayong Zhou:** Conceptualization (lead); writing – review and editing (lead). **Beiwei Zhu:** Funding acquisition (lead); writing – review and editing (supporting).

## CONFLICT OF INTEREST STATEMENT

The authors declare that there are no conflicts of interest.

## ETHICAL APPROVAL

The study was approved by the Ethical Committee of Dalian Polytechnic University (LZ0160258).

## Supporting information


Supplementary Table 1.

## Data Availability

The data used to support the findings of this study can be made available by the corresponding author upon request.
