# Supplementary material for: Trans, trans‐2,4‐decadienal, a lipid peroxidation product, aggravates insulin resistance in obese mice by promoting adipose inflammation
Source: Food Sci Nutr. 2024 Jun 17;12(9):6398–410. doi: 10.1002/fsn3.4273 (PMC11561848; doi:10.1002/fsn3.4273)
Supplement: Supplementary file 1 — Supplementary Table 1. [file FSN3-12-6398-s001.docx]

**Supplementary Table 1.** Composition of HFD and NCD diets.

|  | **HFD (cat# D12492)** | **NCD (cat# D12450J)** |
| --- | --- | --- |
| **Nutritional facts** | kcal% | kcal% |
| Protein | 20 | 20 |
| Carbohydrate | 20 | 70 |
| Fat | 60 | 10 |
| **Ingredients** | g | g |
| Casein | 200 | 200 |
| L-cysteine | 3 | 3 |
| Corn starch | 0 | 506.2 |
| Maltodextrin | 125 | 125 |
| Sucrose | 72.8 | 72.8 |
| Cellulose (Solka Floc/FCC200) | 50 | 50 |
| Lard | 245 | 20 |
| Soybean Oil (USP) | 25 | 25 |
| S10026B (Minerals) | 50 | 50 |
| Choline tartrate (vitamin) | 2 | 2 |
| V10001C (vitamin) | 1 | 1 |
| Dye, Bule, FD＆C #1 | 0.05 | 0.01 |
| Dye, Yellow, FD＆C #5 | 0 | 0.04 |

**Supplementary Table 2.** Lipid composition of corn oil.

| **Composition** | **Content** |
| --- | --- |
| Brassicasterol | ≤0.3% |
| Fatty Acid (<C14) | ≤0.1% |
| Fatty Acid (C14) | ≤0.1% |
| Fatty Acid (C16) | 8.6 - 16.5 % |
| Fatty Acid (C16:1) | ≤0.5% |
| Fatty Acid (C18) | 1.0 - 3.3% |
| Fatty Acid (C18:1) | 20.0 - 42.2% |
| Fatty Acid (C18:2) | 39.4 - 62.0% |
| Fatty Acid (C18:3) | 0.5 - 1.5% |
| Fatty Acid (C20) | ≤0.8% |
| Fatty Acid (C20:1) | ≤0.5% |
| Fatty Acid (C22) | ≤0.3% |
| Fatty Acid (C22:1) | ≤0.1% |
| Fatty Acid (C24) | ≤0.4% |
